# The Mental Hospital Service

**Published:** 1914-03-21

**Authors:** 


### The Mental Hospital Service.

### ASSOCIATION OF ASSISTANT MEDICAL OFFICERS.

A general meeting of members of the newly-formed Association of Assistant Medical Officers of Mental Hospitals was held last Saturday at Westminster Palace Hotel to receive a report concerning the steps already taken to improve the status of the service and to discuss future plans. Dr. Dunn was elected to the chair.

The Hon. Secretary (Dr. Stuart) reported that it was proposed to hold a Conference of Assistant Medical Officers at Durham later in the year. On January 3 last the Association was formed, a code of rules was drawn up and adopted, and an executive committee chosen. The offer of the Status Committee of the Medico-Psychological Association of Great Britain and Ireland to co-opt on to that body a member of the Association had been complied with. The adoption of the report was moved by Dr. Erskine, seconded by Dr. Maule Smith, and carried.

Dr. Stuart then read a report of the interview which Dr. Dunn, Dr. G. F. Barham, Dr. Shaw, and himself had with the Commissioners in Lunacy on the 4th inst., when fourteen members of the board were present. The letter sent to the various visting committees used as text, set forth consultation with the executive of the Association as well as with other bodies when legislation concerning lunacy was contemplated; the right of assistant medical officers to be married and have a separate house as quarters, and the equal right of such officer, even if single, to have such quarters that he could ask a lady relative to make a short stay. It was also asked that study-leave be granted.

The Chairman said the deputation were very cordially received by the Commissioners. The Commissioners said a feeling of delicacy had caused them to abstain from doing much inspection of officers' quarters, but they proposed to do so. In asking for study-leave the idea was to become versed in modern methods of carrying out tests appertaining to bedside work. And there should be a laboratory provided; at present, in many asylums, such processes had to be carried out in the surgery, where one was liable to frequent interruption.

In the ensuing general discussion it was stated that salary increases was not made a special point of at the interview, as the Commissioners were scarcely the body to intervene effectively in that respect. There did not appear to be more than fourteen assistant medical officers of mental hospitals living in houses. There was evidence of an awakened interest in the matter, and in Norfolk a sub-committee had been appointed to consider the matter. With regard to the non-use of some of the provided laboratories, a member thought the authorities should appoint men capable of working them. Study-leave should not be expected by a new entrant to the service. Members urged that the matter should be placed strongly before the proposed Durham Conference, and eventually it was unanimously agreed that the question of study-leave be left to the new executive to deal with as they thought fit. The report of the deputation was approved. The balance sheet showed a membership of 130.

